# Balancing Conformity and Low-Dose Brain Exposure Across Gamma Knife and Linac-Based Stereotactic Radiosurgery Techniques for Multiple Brain Metastases

**DOI:** 10.3390/cancers18071113

**Published:** 2026-03-30

**Authors:** Cristina Teixeira, Orbay Askeroğlu, Marlies Boussaer, Sven Van Laere, Selçuk Peker, Mark De Ridder, Thierry Gevaert

**Affiliations:** 1 Department of Radiotherapy, Research Centre for Digital Medicine, UZ Brussel, Vrije Universiteit Brussel, 1090 Brussels, Belgium; cristina.ferroteixeira@uzbrussel.be (C.T.); marlies.boussaer@uzbrussel.be (M.B.); mark.deridder@uzbrussel.be (M.D.R.); 2 Department of Neurosurgery, Gamma Knife Center, Koç University Hospital, Istanbul 34025, Turkeypeker@selcukpeker.com (S.P.)

**Keywords:** stereotactic radiosurgery, treatment techniques, brain metastasis, conformity index, low-dose spread

## Abstract

**Background/Objectives**: LINAC-based single-isocenter (SIT) stereotactic radiosurgery (SRS) enables efficient treatment of multiple brain metastases but may compromise target conformity and increase low-dose brain exposure, particularly for spatially distributed lesions. Dual-isocenter techniques (DITs) may mitigate these limitations, while Gamma Knife (GK) remains the reference standard for high-selectivity radiosurgery. This study compares SIT- and DIT LINAC-based SRS with GK, focusing on target conformity and low-dose brain exposure under equivalent, zero-margin targeting assumptions. **Methods**: Twenty-eight patients with multiple brain metastases (197 lesions) were included in this retrospective planning study. For each patient, three plans were generated: a GK plan and LINAC-based SIT and DIT plans using automated dynamic conformal arc optimization (Elements Multiple Brain Metastases). All plans were generated using a zero-millimeter GTV-to-PTV margin strategy. For DIT, lesions were automatically clustered and assigned to two isocenters. Target coverage required ≥99% of each GTV to receive the prescription dose. Plan quality was evaluated using the Paddick Conformity Index (PCI) on a per-lesion basis and low-dose brain volumes (V12, V10, V5, V4, and V3 Gy) on a per-patient basis. Paired non-parametric tests and multivariable models were used to assess technique-related differences and associations with total target volume and lesion count. **Results**: GK achieved the highest median PCI (0.83), followed closely by DIT (0.77), while SIT plans demonstrated significantly lower conformity (0.73). Compared with GK, the median PCI difference was −0.05 for DIT and −0.08 for SIT. Conformity for DIT remained stable across lesion volumes and lesion counts, whereas GK conformity increased modestly with lesion size. Low-dose brain exposure differed significantly between techniques at all dose levels (*p* < 0.001). GK consistently yielded the lowest Vx volumes, SIT the highest, and DIT intermediate values. Relative to GK, SIT plans showed progressively larger increases in low-dose exposure at lower dose levels (mean ΔV3 ≈ +149 cc), while DIT reduced this low-dose spread (mean ΔV3 ≈ +117 cc). Total target volume was the dominant predictor of low-dose brain exposure across all techniques, with a smaller additional contribution from lesion count. **Conclusions**: DIT LINAC-based SRS significantly improves target conformity and reduces low-dose brain exposure compared with SIT delivery, achieving dosimetric performance that closely approximates Gamma Knife under equivalent zero-margin targeting assumptions. While Gamma Knife remains the reference standard for low-dose sparing, dual-isocenter planning represents a clinically robust and scalable alternative that effectively balances plan quality and treatment efficiency in patients with multiple brain metastases.

## Simple Summary

This study aimed to compare target conformity and low-dose brain exposure between Gamma Knife and LINAC-based single- and dual-isocenter stereotactic radiosurgery using a zero-millimeter margin strategy enabled by high-precision image guidance and intrafraction monitoring. Dual-isocenter LINAC-based planning improved conformity and reduced low-dose brain exposure compared with single-isocenter delivery, achieving dosimetric performance that approximates Gamma Knife while remaining clinically feasible for patients with multiple brain metastases.

## 1. Introduction

Stereotactic radiosurgery (SRS) for the management of brain metastases enables delivery of high ablative doses with submillimetric precision while minimizing irradiation of surrounding normal brain tissue [1]. Since its introduction, Gamma Knife (GK) radiosurgery has been regarded as a reference standard for intracranial SRS owing to its lesion-specific isocentric design and highly non-coplanar beam geometry, which provide steep dose gradients and excellent conformity [2,3]. In the GK paradigm, each metastasis is treated with a dedicated isocenter, resulting in highly conformal dose distributions and favorable normal brain sparing, albeit often at the expense of prolonged treatment times in patients with multiple lesions [3,4,5].

Advances in linear accelerator (LINAC) technology, including high-resolution multi-leaf collimators (MLCs), frameless image guidance, and non-coplanar delivery, have enabled single-isocenter, multi-target (SIT) LINAC-based SRS as a time-efficient alternative for patients with multiple brain metastases. By treating all targets simultaneously from a common isocenter, SIT approaches substantially reduce treatment time and delivery complexity compared with multi-isocenter techniques [6,7,8,9]. LINAC-based SIT SRS is commonly delivered using volumetric modulated arc therapy (VMAT) or dynamic conformal arc therapy (DCA). While VMAT offers high modulation flexibility and efficient delivery, inverse-optimized DCA has been shown to achieve steeper dose fall-off and reduced intermediate-dose spillage in multi-lesion SRS [10,11,12,13]. However, as the number and spatial extent of metastases increase, intrinsic geometric limitations of single-isocenter planning become increasingly apparent. A key limitation is island blocking, in which overlapping projections of spatially separated targets within a beam’s-eye view restrict optimal MLC shaping, resulting in compromised conformity and increased low- and intermediate-dose exposure to normal brain tissue [14]. Multiple studies have demonstrated that with increasing lesion number and spatial dispersion, maintaining favorable conformity indices, dose gradients, and low-dose sparing become progressively more challenging using SIT approaches, independent of delivery technique [15,16,17].

Dedicated planning systems have been developed to automate SIT planning and reduce some of these limitations [18]. Elements Multiple Brain Metastases (MBM) (Brainlab, Munich, Germany) integrates automated isocenter placement, arc selection, and inverse-optimized DCA planning within a standardized workflow [19]. By enabling extensive non-coplanar arc sampling and intelligent beam-to-lesion assignment, the software improves conformity, mitigates island blocking, and reduces unnecessary low-dose spread compared with conventional SIT techniques [20]. Nevertheless, even with automation, fundamental geometrical constraints inherent to single-isocenter delivery persist when lesions are widely distributed. To address these limitations, several groups have explored multi-isocenter strategies, typically employing two to three isocenters to group lesions based on spatial proximity or hemispheric location [21,22,23]. These approaches have been shown to improve conformity and dose gradients compared with single-isocenter techniques, particularly for spatially dispersed metastases. However, most reported implementations rely on manual isocenter placement, predefined arc arrangements, and limited non-coplanar sampling, increasing planner workload and limiting clinical scalability. VMAT-based multi-isocenter solutions continue to face trade-offs between plan quality, normal brain sparing, and treatment efficiency.

These considerations have motivated interest in combining the dosimetric advantages of limited lesion partitioning with the efficiency and reproducibility of automated planning, while avoiding the logistical burden of multiple isocenter setups. The automation and near-4π non-coplanar optimization capabilities of Elements MBM provide a framework in which a two-isocenter strategy may offer an optimal balance between plan quality, normal brain sparing, and clinical feasibility. In contemporary LINAC-based stereotactic workflows employing high-precision image guidance and intrafraction monitoring, margin-free (0 mm) target definitions can be applied with submillimetric accuracy comparable to dedicated cranial platforms, enabling fair dosimetric comparison across delivery techniques [24,25,26].

Accordingly, the aim of this study is to evaluate the dosimetric performance of an automated dual-isocenter (DIT) LINAC-based SRS strategy relative to SIT LINAC-based SRS and Gamma Knife radiosurgery. A dual-isocenter approach may improve target coverage and dose conformity compared with a single-isocenter technique, while maintaining a clinically practical workflow and avoiding the increased treatment time and complexity associated with three or more isocenters. By focusing on target conformity and low-dose brain exposure, this work seeks to determine whether a clinically feasible two-isocenter strategy can achieve high conformality and efficiency in patients with multiple brain metastases.

## 2. Materials and Methods

### 2.1. Patient Selection

This retrospective planning study included 28 patients with multiple brain metastases treated clinically with LINAC-based stereotactic radiosurgery on a Varian TrueBeam STx system (Varian, Palo Alto, CA, USA). Patients had between 4 and 14 brain metastases for a total of 197 lesions. All patients underwent high-resolution contrast-enhanced magnetic resonance imaging (MRI) for stereotactic targeting and a computed tomography (CT) for dose computation. To enable a uniform and contemporary comparison, all LINAC-based plans, regardless of the software version used at the time of clinical treatment, were retrospectively replanned. For each patient, three plans were generated and analyzed: a GK radiosurgery plan that is seen as the reference plan, a LINAC-based SIT multi-target plan, and a DIT LINAC-based multi-target plan (DIT) using Elements MBM version 4.5.

### 2.2. Dose Prescription and Dosimetric Evaluation

All plans were aimed at achieving coverage of 99% for each GTV by the prescription dose 20 Gy, which is to be identical across GK, SIT, and DIT plans for each patient to allow for direct dosimetric comparison while also allowing intralesional dose heterogeneity to be consistent with standard stereotactic radiosurgery practice. PTV margin was omitted as comparable treatment accuracy is achieved.

The plan quality was evaluated using the target coverage dose of 99% for the GTV (D99%), as well as Paddick Conformity Index (PCI) [27] and volumes of normal brain minus GTV receiving 12 Gy (V12), 10 Gy (V10), 5 Gy (V5), and 3 Gy (V3).

In cases where closely spaced metastases resulted in unavoidable prescription isodose bridging, individual lesion separation was not enforced. In these instances, the metastases involved were considered as a combined target volume for conformity analysis, and the PCI was calculated with respect to the summed GTV volume encompassed by the shared prescription isodose.

All organ-at-risk doses were required to meet standard single-fraction stereotactic radiosurgery constraints.

### 2.3. Gamma Knife Reference Plans

Gamma Knife reference plans were generated retrospectively using the Leksell Gamma Knife Esprit system (Elekta AB, Stockholm, Sweden) with the GammaPlan v11.4.2 Lightning inverse planning algorithm. Lightning employs automated inverse optimization to determine shot placement, collimator size selection (4, 8, and 16 mm), sector weighting, and shot weighting with the objective of maximizing target conformity while minimizing dose to the surrounding normal brain tissue.

Each metastasis was treated using an individual isocenter, consistent with standard Gamma Knife practice. For each target, Lightning automatically optimized the number and spatial distribution of shots to achieve the prescribed dose coverage while maintaining steep dose gradients. Collimator sizes and sector blocking were adaptively selected per shot to conform the prescription isodose to the target geometry and to limit dose spill to adjacent normal brain tissue. In cases where targets were closely spaced and unavoidable prescription isodose bridging occurred, the involved metastases were treated as a composite target volume for conformity analysis.

Intralesional dose heterogeneity was allowed in accordance with standard Gamma Knife stereotactic radiosurgery practice. Normalization and shot weighting were automatically adjusted by the Lightning optimizer to maximize conformity while minimizing intermediate- and low-dose exposure outside the target volumes.

Plan quality was reviewed to ensure compliance with institutional standards for target coverage and organ-at-risk dose constraints.

### 2.4. LINAC-Based Planning

LINAC-based stereotactic radiosurgery plans were generated using a dedicated automated multiple brain metastases planning workflow Elements MBM, based on inverse optimization of DCA with extensive non-coplanar beam sampling. The automated algorithm simultaneously optimizes isocenter placement, arc geometry, gantry and couch angles, collimator rotation, MLC shaping, and arc weighting with the objective of maximizing target conformity while minimizing dose to normal brain tissue.

Plans were made using an automated protocol employing 4–7 non-coplanar couch angles, with 2–4 dynamic conformal arcs per couch position, depending on lesion number and spatial distribution. Arc start and stop angles were automatically adjusted during optimization to avoid opposing arcs and to reduce unnecessary normal tissue irradiation. Collimator rotations were varied across arcs to mitigate interleaf leakage effects and to improve dose conformity. For each arc, MLC leaf pairs were constrained to irradiate only a single lesion at any given beam’s eye view, thereby preventing island blocking and limiting low-dose exposure between spatially separated targets, as described previously for automated DCA-based multiple metastases planning.

Target optimization objectives for each GTV and conformity optimized on a per-lesion basis using the Conformity Index counted as an internal objective function. Normal brain sparing was achieved by minimizing dose spillage outside the target volumes, with particular emphasis on reducing intermediate- and low-dose exposure.

In the SIT plans, all lesions were treated simultaneously from a single common isocenter automatically placed at the geometric center of mass of all targets. All arcs were optimized to collectively irradiate the full set of lesions while respecting the constraint that each MLC leaf pair could expose only one target per arc.

In the DIT plans, lesion grouping was performed manually by experienced stereotactic planners to reflect a reproducible real-world workflow rather than relying on a treatment planning system–embedded automated clustering tool. Clustering was based primarily on geometric proximity and the three-dimensional spatial distribution of metastases within the brain. Lesions in close spatial proximity were preferentially assigned to the same isocenter to minimize the maximum lesion-to-isocenter distance and thereby reducing the chance of being in the 5 mm leaves of the MLC. When feasible, lesions were distributed in a balanced manner between the two isocenters to avoid excessive modulation complexity at a single isocenter; however, geometric compactness was prioritized over strict numerical symmetry. Lesions separated by larger distances or located in distinct hemispheric or axial regions were preferentially allocated to different isocenters to limit low-dose spread and improve conformity. The isocenter for each cluster was subsequently positioned at the geometric centroid of the grouped lesions, resulting in two isocenters per patient. For each isocenter, an independent set of non-coplanar dynamic conformal arcs was generated using the same automated optimization strategy as for the single-isocenter approach. This approach was designed to improve conformity and dose gradients for spatially distributed lesions while maintaining clinical feasibility by limiting the number of isocenter setups.

All plans underwent systematic manual review and, when necessary, refinement to improve conformity and normal brain sparing while maintaining target coverage and OAR compliance. Manual refinements included helping structures, selective modification of dynamic conformal arc (DCA) complexity, and, where permitted by the software, the addition of supplementary arc passages. Due to software limitations, a maximum of three lesions could be actively optimized per isocenter for extra arc passages; therefore, up to three additional arc groupings were introduced when required to optimize spatially complex or widely separated lesions. These adjustments were performed on a per-patient and per-isocenter basis to achieve the most favorable balance between conformity and normal brain sparing.

### 2.5. Statistical Analysis

Lesion-level and patient-level analyses were performed separately. Continuous variables are reported as median (interquartile range) or mean ± standard deviation, where appropriate. All tests were two-sided, with *p* < 0.05 considered statistically significant. Lesion-level conformity was compared between techniques using paired Wilcoxon signed-rank tests. Associations between lesion volume and PCI were assessed using Spearman correlation. A linear mixed-effects model with patient as a random effect was used to evaluate the association between treatment technique and conformity while adjusting for lesion volume. Low-dose brain exposure (V12Gy–V3Gy) was analyzed at the patient level. Differences between techniques were assessed using the Friedman test with post hoc paired Wilcoxon testing. Determinants of low-dose exposure were evaluated using Spearman correlation and multivariable linear regression including treatment technique, total target volume, and number of metastases. All analyses were performed using SPSS software v27.0. All analyses were performed using SPSS software. Regression results are reported as unstandardized beta coefficients (B) with corresponding confidence intervals. Correlation analyses are presented using Spearman’s rho (ρ).

## 3. Results

**Figure 1:**
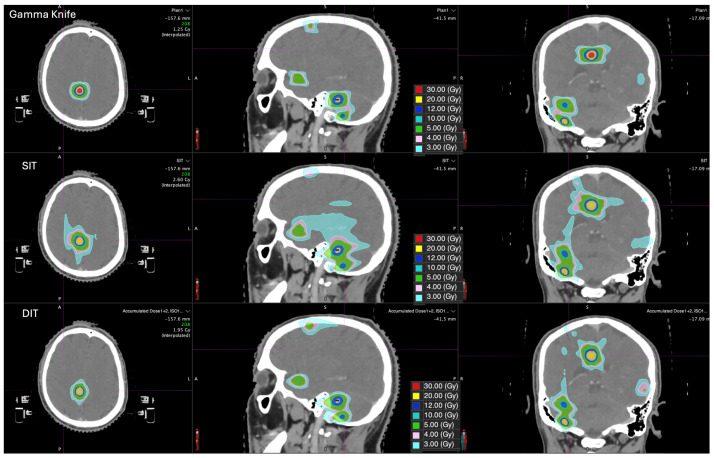
Typical dose distribution Gamma Knife, single-isocenter technique (SIT) and dual-isocenter technique (DIT) for a patient with 6 brain metastases. Identical dose color scales and isodose levels are used across the three techniques.

**Figure 2:**
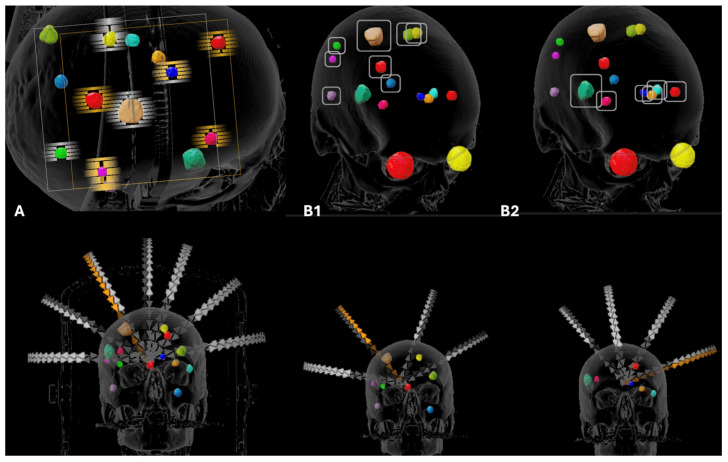
Patient with 10 lesions. (**A**). Single-isocenter technique with island blocking. (**B1**). Arc arrangement and isocenter 1 placement. (**B2**). Arc arrangement and isocenter 2 placement.

Twenty-eight patients with a total of 197 metastatic lesions were analyzed. Each patients had a high median number of metastases and a wide range of total target volumes, allowing evaluation of plan performance across varying tumor burdens. The median lesion volume was 0.53 cc (interquartile range [IQR] 0.27–1.04 cc). All patients were planned with GK, SIT LINAC-based, and DIT LINAC-based techniques, enabling paired comparisons. Prescription isodose bridging occurred for SIT in 6 of 28 patients (21%), involving 17 of 197 metastases (8.6%). Figure 1 shows a typical dose distribution of Gamma Knife, SIT, and DIT. Figure 2 shows an automated single isocenter arc arrangement with island blocking. (A) and a dual-isocenter (B1–B2) arc arrangement and isocenter placement for a patient with 10 brain lesions.

### 3.1. Paddick Conformity Index (PCI)

Median PCI was highest for GK plans (0.83, IQR 0.78–0.85), followed by DIT plans (0.77, IQR 0.73–0.81) and SIT plans (0.73, IQR 0.69–0.79).

On a per-lesion paired analysis, both SIT and DIT showed significantly lower conformity compared with GK. The median difference in PCI was −0.08 for SIT plans and −0.05 for DIT plans (Wilcoxon signed-rank test).

A moderate positive correlation between lesion volume and PCI was observed for GK plans (Spearman ρ = 0.31, *p* < 0.001), whereas no significant correlation was found for SIT or DIT techniques.

In a mixed-effects model with patient as a random effect and correction for lesion volume, both SIT (β = −0.084 ± 0.009, t = −9.6, *p* < 0.001) and DIT (β = −0.033 ± 0.005, t = −5.1, *p* < 0.001) remain independently associated with lower conformity compared with GK. The number of lesions per patient was not significantly associated with conformity.

### 3.2. Low-Dose Exposure of Normal Brain Tissue

Low-dose brain exposure was analyzed at the patient level (n = 28). Across all evaluated dose thresholds (V12Gy–V3Gy), significant differences between techniques were observed (Friedman test with post hoc paired testing).

GK consistently achieved low Vx volumes, whereas SIT plans resulted in the largest low-dose volumes and DIT plans showed intermediate values.

Compared with GK, SIT plans demonstrated significantly higher V12Gy and V10Gy volumes (mean Δ+3.93 ± 2.1 cc and +6.53 ± 3.4 cc, respectively; both *p* < 0.001), with progressively larger differences at lower dose levels (V5Gy: +48.18 ± 22.6 cc; V4Gy: +85.98 ± 41.3 cc; V3Gy: +149.26 ± 69.8 cc; all *p* < 0.001). DIT plans showed significantly lower Vx volumes compared to SIT and smaller differences in Vx compared to GK.

Spearman correlation analysis demonstrated a strong positive association between total target volume and Vx across all techniques and dose levels (ρ ≈ 0.75–0.85, all *p* < 0.001). Associations with the number of metastases were weaker (ρ ≈ 0.05–0.20), indicating that tumor burden rather than lesion count was the dominant determinant of low-dose brain exposure.

Multivariable linear regression models including treatment technique, total target volume, and number of metastases confirmed that total target volume was the strongest independent predictor of low-dose brain exposure across all dose thresholds (all *p* < 0.001). After adjustment for these factors, DIT techniques remained independently associated with higher Vx volumes compared with GK.

## 4. Discussion

An important consideration in interpreting the present findings is that all plans were generated using a zero-millimeter GTV-to-PTV margin strategy across Gamma Knife, single-isocenter, and dual-isocenter LINAC-based techniques. While this approach may differ from institutional practices that apply a 1 mm margin, it reflects our current clinical workflow when dedicated measures are implemented to minimize geometric uncertainty. These include strict control of MRI distortion [28], high-resolution image guidance and six-degree-of-freedom couch corrections [24,29], image guidance at each couch angle, and intrafraction verification with a tolerance threshold of 0.5 mm/0.5° [25]. Additionally, mechanical couch stability is monitored throughout treatment delivery, and intra-arc imaging further reduces the risk of unrecognized displacement or patient movement [26].

Under these controlled conditions, frameless LINAC-based stereotactic radiosurgery can achieve submillimetric geometric accuracy comparable to dedicated cranial platforms such as Gamma Knife. Although the use of a zero-margin strategy requires rigorous quality assurance and may not be universally applicable, the residual geometric uncertainty in this setting is minimal, and its dosimetric impact, even for lesions located further from the isocenter is expected to be negligible [26,30].

This design choice differs from several comparative studies that have applied uniform margin expansions to LINAC-based plans, most commonly 1 mm, while omitting margins for Gamma Knife radiosurgery. Marianayagam et al. [31] applied a 1 mm GTV-to-PTV expansion for LINAC-based single-isocenter plans while maintaining zero-margin planning for Gamma Knife, inherently biasing conformity and low-dose metrics against LINAC-based approaches. While such assumptions may reflect local practice patterns, they do not universally apply to modern LINAC-based SRS workflows employing high-resolution image guidance, intrafraction monitoring, and robust geometric validation [24,25,26,28].

Multiple contemporary studies have demonstrated that, when these measures are employed, dosimetric equivalence at the target level between Gamma Knife and LINAC-based SRS can be achieved without systematic margin expansion, supporting the validity of a zero-margin strategy for comparative dosimetric evaluations [3]. Accordingly, the primary objective of the present analysis was not to reassess targeting accuracy or geometric precision, but rather to evaluate how different delivery strategies, single-isocenter versus dual-isocenter LINAC-based SRS, compare with Gamma Knife when target coverage and margins are held constant. By focusing on target conformity and low-dose brain exposure under equivalent targeting assumptions, this study isolates the impact of delivery geometry and beam arrangement, providing a clearer assessment of the trade-offs inherent to each technique.

This study therefore provides a comprehensive comparison of target conformity and low-dose normal brain exposure between Gamma Knife and contemporary LINAC-based stereotactic radiosurgery strategies in patients with multiple brain metastases. By integrating lesion-level conformity analysis with patient-level low-dose metrics and multivariable modeling, we demonstrate that technique-related differences persist independently of tumor burden and lesion count and are primarily driven by planning geometry and delivery strategy, consistent with prior dosimetric analyses [7,14].

The inferior conformity observed with single-isocenter plans is consistent with well-described geometric and dosimetric limitations of treating spatially distributed targets from a single common isocenter. These include island blocking, increased sensitivity to rotational uncertainties, and reduced flexibility in shaping dose to individual lesions [14,15]. The MBM platform uses automated dynamic conformal arc optimization, in which individual metastases are selectively irradiated at each control point, thereby minimizing open MLC regions between spatially separated targets and substantially reducing the classical island blocking effect. Therefore, the lower PCI observed for the single-isocenter technique is unlikely to be primarily caused by residual island blocking. Rather, the reduced conformity more likely reflects inherent geometric limitations of the single-isocenter paradigm. When multiple lesions with varying sizes and spatial separation are treated around a common isocenter, off-axis MLC projection effects and the need to balance aperture shaping across heterogeneous targets within shared arcs can compromise conformity. This is consistent with previous evaluations of single-isocenter approaches, including MBM-based planning, which report decreasing conformity with increasing inter-lesion distance and geometric complexity compared to multi-isocenter strategies [5,8].

In contrast, dual-isocenter planning substantially alleviates these constraints by introducing limited lesion partitioning while maintaining automated optimization and non-coplanar beam sampling. This resulted in a marked improvement in conformity relative to single-isocenter approaches and conformity values that approximate those achieved with Gamma Knife. Importantly, dual-isocenter planning consistently narrowed the conformity gap relative to Gamma Knife across a wide range of lesion volumes and lesion counts. While small numerical differences in conformity remained detectable, their magnitude was limited and of uncertain clinical relevance. Modest conformity variations remains uncertain, particularly when established normal-brain dose constraints are respected. Aiyama et al. [32] demonstrated that neither conformity index nor gradient index independently correlated with complication risk following SRS, with toxicity being more strongly associated with peripheral dose parameters and V12Gy [33]. Furthermore, improvements in conformity metrics have not consistently translated into superior clinical outcomes. Taken together, these data suggests that conformity indices alone are imperfect surrogates for clinical effect. Therefore, the residual PCI differences observed between dual-isocenter LINAC and Gamma Knife are more likely reflective of intrinsic platform geometry and beam delivery characteristics rather than clinically meaningful divergence in expected local control or toxicity outcomes. Within the context of controlled normal-brain dose exposure, the observed differences are unlikely to translate into measurable clinical impact. In this context, dual-isocenter planning can be considered non-inferior to Gamma Knife with respect to target conformity, particularly when balanced against the potential advantages in treatment efficiency and clinical workflow for patients with multiple metastases.

Target volume was weak but significantly associated with conformity for Gamma Knife plans, with slightly higher conformity observed for larger lesions. This relationship was not observed for either single- or dual-isocenter LINAC-based techniques, suggesting that LINAC-based conformity is less dependent on lesion size and more strongly influenced by geometric configuration and planning strategy. Notably, conformity for dual-isocenter plans remained stable across the full range of target volumes analyzed, further supporting the robustness and scalability of this approach.

The absence of an independent association between the number of lesions and conformity underscores that high plan quality can be maintained in patients with extensive intracranial disease, provided that appropriate planning strategies are employed. This finding is particularly relevant for dual-isocenter planning, which maintained near-Gamma Knife conformity even in patients with a high lesion burden, supporting its feasibility in complex multi-lesion scenarios.

Beyond target conformity, low-dose exposure of normal brain tissue represents a critical consideration in stereotactic radiosurgery, given its potential association with neurocognitive toxicity and radionecrosis [33]. In the present study, total target volume emerged as the dominant determinant of low-dose brain exposure across all techniques, a finding that aligns with recent clinical outcome data. In a large retrospective cohort of patients with four or more brain metastases treated with single-fraction SRS, Parikh et al. [34] demonstrated that total lesion volume, but not composite V12Gy, was predictive of clinically relevant late radiation adverse events, highlighting the central role of tumor burden in determining normal brain risk. These findings support the interpretation that volumetric disease burden, rather than isolated dosimetric thresholds, is the primary driver of low-dose exposure and its potential clinical consequences in the multi-metastatic setting. Nevertheless, even after adjustment for total target volume and number of metastases, treatment technique remained an independent predictor of low-dose exposure, indicating that differences between systems reflect intrinsic geometric and delivery-related characteristics rather than patient or disease factors alone.

Single-isocenter LINAC-based plans were associated with a substantial and progressively increasing low-dose brain exposure relative to Gamma Knife, particularly at lower dose thresholds (V5–V3Gy). This behavior reflects the broader low-dose bath inherent in single-isocenter delivery for spatially distributed targets. Dual-isocenter planning consistently mitigated this effect, yielding low-dose brain volumes that were markedly closer to those of Gamma Knife and substantially reduced compared with single-isocenter approaches across all evaluated dose levels. Although small absolute differences in low-dose metrics remained, these differences must be interpreted in the context of clinical feasibility, treatment time, and patient throughput, particularly in patients with multiple metastases.

An important enabling factor of the present study is the use of a dedicated automated planning platform for both single- and dual-isocenter LINAC-based stereotactic radiosurgery. Planning SRS for multiple brain metastases is inherently complex, with plan quality highly dependent on planner experience and institutional practice. Previous studies have demonstrated substantial inter-planner variability in conformity and low-dose metrics for LINAC-based SRS, even when standardized planning protocols are applied [20]. Automated planning solutions have been shown to reduce this variability and to deliver consistent, high-quality plans across institutions. Automated multiple brain metastases planning has achieved conformity and low-dose characteristics comparable to dedicated radiosurgery platforms while substantially improving reproducibility and planning efficiency. In this context, automation was essential to enable a fair and reproducible comparison between single- and dual-isocenter strategies. By applying identical automated optimization principles to both approaches, the present study isolates the impact of delivery geometry and isocenter configuration, rather than planner-dependent factors, and demonstrates the clinical feasibility of dual-isocenter planning. A dual-isocenter linac plan requires approximately twice the beam-on time of a single-isocenter plan, as arcs are delivered sequentially per isocenter. In our workflow, the additional time increase was primarily due to couch repositioning, additional image guidance verification, and arc delivery repetition. However, even with this increase, total treatment time remains substantially shorter than Gamma Knife in most clinical scenarios. For Gamma Knife, treatment duration is strongly dependent on number of lesions, number of shots per lesion and source activity (decay of Co-60). Chea et al. [5] found a factor 7 difference between Gamma Knife beam-on time and SIT, proving that DIT approach represents a clinically scalable compromise between plan quality and efficiency.

Expanding the number of isocenters in the treatment of multiple brain metastases requires careful balance between potential gains in conformity and the accompanying increase in planning complexity and treatment time. While additional isocenters may theoretically offer greater geometric flexibility for widely separated lesions, the literature largely focuses on single- and dual-isocenter strategies, with limited evidence demonstrating consistent added benefit from routinely using three or more isocenters in modern automated workflows. Available data indicate that transitioning from one to two isocenters improves conformity, whereas further increases do not appear to yield proportional gains and may unnecessarily increase complexity and delivery time [7,8,21,35,36].

Unlike earlier dual-isocenter approaches that relied on predefined configurations and substantial manual optimization, the software platform used in this study applies automated inverse optimization to determine isocenter placement and beam geometry with the goal of maximizing conformity and minimizing dose spillage. This enhances standardization while preserving limited planner refinement when needed. Within this context, the use of two isocenters represents a pragmatic choice to improve conformity and reduce low-dose spread without compromising efficiency.

Important note is that no automated TPS-driven clustering algorithm was used, as the aim was to determine whether a structured, planner-driven geometric grouping strategy could yield meaningful dosimetric improvements over a single-isocenter approach while remaining clinically feasible. To evaluate the robustness of this manual strategy, we retrospectively compared our grouping decisions with established clustering frameworks described in the literature, including the agglomerative and k-means approaches reported by Hui et al. [37]. These methods aim to minimize isocenter–target distance using minimax or sum-of-squares objectives to reduce rotational uncertainty. We observed strong concordance between our manually generated clusters and those predicted by formal geometric algorithms, particularly with respect to minimizing maximum isocenter–target distance. This suggests that experienced planner-driven grouping inherently approximates established mathematical clustering principles, supporting the validity and scalability of the manual dual-isocenter workflow in routine clinical practice.

## 5. Conclusions

Under a zero-margin delivery strategy dual-isocenter linac-based stereotactic radiosurgery substantially improves target conformity and low-dose brain sparing compared with single-isocenter approaches and achieves dosimetric performance that assesses that of Gamma Knife radiosurgery. The conformity for dual-isocenter plans remained stable across a wide range of lesion volumes and lesion number, supporting the robustness of this approach in patients with multiple brain metastases. Gamma Knife has long been regarded as the reference standard for intracranial stereotactic radiosurgery; however, dual-isocenter planning represents a clinically meaningful and scalable alternative, particularly for patients with multiple brain metastases, where treatment time, workflow efficiency, and logistical considerations are relevant.

Total target volume was the dominant determinant of low-dose brain exposure, with lesion count providing an additional but smaller contribution, underscoring the importance of individualized planning strategy selection.

## Data Availability

No new data were created or analyzed in this study. Data sharing is not applicable to this article.
